# Prevalence, Comparative Risk, and Predictors of Shock in Children With Multisystem Inflammatory Syndrome Associated With COVID‐19: A Systematic Review and Meta‐Analysis

**DOI:** 10.1111/jpc.70470

**Published:** 2026-06-10

**Authors:** Asif Jan, Bushra Waheed, Ghadah Ali Hussein, Pratima Pandey, Huma Sultana, Shubham Kumar, Mequanente Dagnaw

**Affiliations:** ^1^ Department of Pharmacy University of Peshawar Peshawar KP Pakistan; ^2^ Saidu Group of Teaching Hospitals Saidu Sharif Swat KP Pakistan; ^3^ District Headquarter Hospital Charsadda Charsadda KP Pakistan; ^4^ Department of Pharmacy University of Swabi Swabi KP Pakistan; ^5^ College of Medical and Health Technologies, Al‐Zahraa University for Women Karbala Iraq; ^6^ Department of Pharmacy Practice National Institute of Pharmaceutical Education and Research S.A.S. Nagar India; ^7^ Department of Pharmaceutical Chemistry Faculty of Pharmacy, MS Ramaiah University of Applied Sciences Bangalore India; ^8^ Dental Research Cell, Dr. D. Y. Patil Dental College & Hospital, Dr. D. Y. Patil Vidyapeeth (Deemed To Be University) Pune India; ^9^ Global Research Cell, Dr. D. Y. Patil Medical College Hospital and Research Centre, Dr. D. Y. Patil Vidyapeeth, (Deemed To Be University) Pune India; ^10^ Department of Epidemiology and Biostatistics Institute of Public Health, University of Gondar Gondar Ethiopia; ^11^ Department of Medical Biotechnology Institute of Biotechnology, University of Gondar Gondar Ethiopia

**Keywords:** child health, good health and well‐being, meta‐analysis, multisystem inflammatory syndrome in children, SARS‐CoV‐2, shock

## Abstract

**Background:**

Multisystem inflammatory syndrome in children (MIS‐C) is a severe hyperinflammatory condition associated with SARS‐CoV‐2 infection and is frequently complicated by cardiovascular dysfunction, particularly shock. Early recognition of shock in MIS‐C is critical to reduce morbidity and mortality.

**Objective:**

This systematic review and meta‐analysis aimed to evaluate the prevalence, comparative risk, and predictors of shock among children with MIS‐C, enhancing clinical management and informing future research.

**Methods:**

A systematic search of PubMed, Embase and Web of Science was conducted from database inception to 15 July 2025. Observational studies reporting prevalence, comparative risk or predictors of shock in children with MIS‐C were included. Random‐effects meta‐analysis with Freeman–Tukey double arcsine transformation was used to estimate pooled prevalence with 95% confidence intervals (CI). Relative risks (RR) were pooled for comparative analyses. Subgroup analyses, sensitivity analyses and meta‐regression were performed to explore heterogeneity. Publication bias was assessed using funnel plots and Egger's regression test.

**Results:**

Seventy studies involving 13 263 children with MIS‐C were included. The pooled prevalence of shock was 56% (95% CI: 49%–63%), although substantial heterogeneity was observed (*I* ^2^ = 98.4%). Sensitivity analyses excluding studies with sample sizes < 50 and < 100 participants yielded pooled prevalence estimates of 50% (95% CI: 40%–59%) and 46% (95% CI: 33%–59%), respectively. Subgroup analyses demonstrated variation according to publication year and diagnostic criteria. Children with MIS‐C had a significantly increased risk of shock compared with Kawasaki disease cohorts (RR = 8.52, 95% CI: 1.24–58.41; *p* < 0.05) and acute/severe COVID‐19 cohorts without MIS‐C (RR = 4.42, 95% CI: 2.44–8.02; *p* < 0.001). Echocardiographic abnormalities, myocardial dysfunction, elevated inflammatory markers and acute kidney injury were consistently associated with shock.

**Conclusion:**

The certainty of evidence ranged from very low to low because of substantial heterogeneity, imprecision and methodological variability across studies. Despite these limitations, shock remains a frequent and serious complication of MIS‐C, highlighting the importance of early cardiovascular assessment and timely recognition of high‐risk children.

## 1 Introduction

Multisystem inflammatory syndrome in children (MIS‐C) emerged alongside the COVID‐19 pandemic, presenting a novel and complex challenge in paediatrics [1, 2, 3]. This hyperinflammatory condition affects multiple organ systems, primarily following or coinciding with SARS‐CoV‐2 infection, even if the initial COVID‐19 illness was mild or asymptomatic. MIS‐C typically presents several weeks after confirmed or suspected SARS‐CoV‐2 infection. Children with MIS‐C often experience prolonged fever, gastrointestinal complaints like abdominal pain, vomiting and diarrhoea, along with mucocutaneous involvement manifested as rash and conjunctivitis [4, 5]. A hallmark feature is cardiovascular involvement, with a spectrum of presentations ranging from myocarditis and pericarditis to coronary artery dilatation and, most concerning, shock [6, 7]. The exact aetiology of MIS‐C remains under investigation, but it is believed to be triggered by a dysregulated immune response to SARS‐CoV‐2 infection in genetically predisposed individuals. This dysregulation leads to a cytokine storm characterized by the excessive release of inflammatory mediators [8, 9]. The cytokine storm disrupts vascular integrity, causing capillary leak and hypotension, potentially culminating in shock [10].

Shock is a time‐sensitive medical emergency requiring immediate intervention. In the context of MIS‐C, prompt recognition and management of shock play a vital role in preventing significant morbidity and mortality [7, 11]. Studies suggest a wide range in the reported prevalence of shock in MIS‐C, ranging from 32% to 76%, highlighting its significance as a potential complication [12, 13, 14, 15, 16, 17]. Patients with shock often experience a more severe clinical course, requiring intensive care support and potentially experiencing long‐term sequelae. Understanding the prevalence and risk factors associated with shock, a life‐threatening complication in MIS‐C, is crucial for early identification, risk stratification and prompt intervention to improve patient outcomes.

By identifying children at high risk of developing shock, clinicians can implement closer monitoring and more aggressive treatment strategies. This could lead to earlier intervention and potentially prevent the progression to full‐blown shock. Knowing the risk factors for shock allows for better patient stratification, aiding in resource allocation and tailoring treatment plans to individual patient needs [18]. Identifying risk factors can contribute to the development of prognostic scores to predict which MIS‐C patients are more likely to experience shock. Such scores would facilitate improved clinical decision‐making and risk communication with families. Existing literature highlights the significant clinical burden of shock in MIS‐C. However, there is a lack of comprehensive data on the exact prevalence of shock in this population. Additionally, the risk factors associated with shock development remain incompletely understood.

By aggregating and analysing data from various studies, a systematic review and meta‐analysis enables more accurate estimates of the prevalence of shock among MIS‐C patients. Moreover, it facilitates the identification of risk factors with a higher degree of statistical significance than would be possible through individual studies alone. The primary objective of this review is to elucidate the current understanding of shock in children with MIS‐C by systematically examining its prevalence and associated risk factors. Through this comprehensive analysis, our goal is to enhance clinical management practices and inform future research endeavours to unravel the complexities of this severe complication, thereby paving the way for more effective prevention and treatment strategies.

## 2 Methods

This systematic review and meta‐analysis adhered to ethical principles outlined in relevant guidelines like the Preferred Reporting Items for Systematic Reviews and Meta‐Analyses (PRISMA) statement [19]. Ethical approval was not required for this type of research that relies on published data.

### 2.1 Literature Search

An extensive literature search was conducted across multiple electronic databases. Core resources included PubMed, Embase and Web of Science until 15 July 2025. A search strategy was formulated using a combination of relevant Medical Subject Headings (MeSH) terms and keywords. Examples included: ‘multisystem inflammatory syndrome’, ‘MIS‐C’, ‘shock’, ‘cardiovascular dysfunction’, ‘children’, ‘paediatrics’. Boolean operators (AND, OR and NOT) were strategically combined to create a focused search string that retrieved relevant studies while minimizing the inclusion of irrelevant articles. A manual search of reference lists from pertinent articles and reviews was conducted to identify any additional studies not captured by the electronic database search. The complete search strategy for each database is presented in Table S2.

### 2.2 Inclusion and Exclusion Criteria

Studies enrolling children diagnosed with MIS‐C were included. Studies reported the incidence or investigated the risk factors associated with shock in the context of MIS‐C. The outcomes of interest encompassed both the overall prevalence of shock and the identification of factors that might increase the risk of its development. Observational studies, including cohort studies, case–control studies and cross‐sectional case series, were considered for inclusion. These observational designs are suitable for exploring the relationships between potential risk factors and the occurrence of shock in the context of MIS‐C. Case reports, editorials and letters were excluded as they do not provide the generalizable data necessary for a robust analysis. Studies involving adults or populations other than children were excluded, as were studies not investigating MIS‐C or shock as a complication. Studies with a different research focus fell outside the scope of this review and were excluded, along with animal studies or in vitro studies.

### 2.3 Selection of Studies

All retrieved citations were imported into EndNote. Two independent reviewers screened the titles and abstracts of all identified studies based on the predefined inclusion and exclusion criteria. Any discrepancies arising between reviewers were resolved through discussion or by consulting a third reviewer to reach a consensus. Full‐text articles of studies deemed potentially relevant based on the initial screening were retrieved for further evaluation. The two independent reviewers independently assessed the full‐text articles against the inclusion and exclusion criteria. Again, any disagreements were addressed through discussion or involving a third reviewer.

### 2.4 Data Extraction and Quality Assessment

A standardized data extraction form was developed to record pertinent information from the included studies. This standardized approach ensured consistent data collection across all studies. Two independent reviewers performed data extraction using the pre‐designed data extraction form. The form encompassed details crucial for this review, including study characteristics (author names, publication year and study design), patient characteristics (sample size, age and sex) and outcomes reported. Data on potential risk factors associated with shock development included demographic data (age, sex and ethnicity) and clinical characteristics (symptoms, laboratory findings). The tagging function of Nested‐Knowledge was used for data extraction.

The quality and reliability of the included studies were critically appraised using a validated tool for assessing the risk of bias in observational studies. The Joanna Briggs Institute (JBI) Critical Appraisal Checklist for prevalence studies was employed for quality assessment. The ROBINS‐E tool was used for studies included in risk assessment.

### 2.5 Data Synthesis and Statistical Analysis

Data were synthesized using appropriate quantitative statistical methods. For the primary outcome, the pooled prevalence of shock among children with MIS‐C was estimated using a random‐effects meta‐analysis model with Freeman–Tukey double arcsine transformation to stabilize variance. Pooled prevalence estimates were reported with corresponding 95% CI. For comparative analyses, relative risk (RR) with 95% CI was calculated to compare the risk of shock in MIS‐C patients versus non‐MIS‐C comparator groups [20].

Statistical heterogeneity across studies was assessed using Cochran's *Q* test and quantified using the *I* ^2^ statistic. An *I* ^2^ value greater than 50% was considered indicative of substantial heterogeneity [21, 22]. Because significant clinical and methodological heterogeneity was anticipated across included studies, random‐effects models were used throughout the analyses.

To explore potential sources of heterogeneity, subgroup analyses were performed according to publication year and diagnostic criteria used for MIS‐C classification. Additional subgroup analyses were conducted for comparative studies by separating MIS‐C versus Kawasaki disease cohorts and MIS‐C versus acute/severe COVID‐19 cohorts. Sensitivity analyses were performed by excluding studies with sample sizes fewer than 50 participants and fewer than 100 participants to evaluate the robustness and stability of the pooled prevalence estimates.

Meta‐regression analyses were conducted using mixed‐effects models to investigate the influence of potential moderators on the prevalence of shock among MIS‐C patients. Univariate meta‐regression analyses were initially performed using study‐level covariates including publication year, mean age, proportion of male participants, sample size and diagnostic criteria. Variables identified a priori were subsequently included in multivariable meta‐regression models to assess their independent contributions to between‐study heterogeneity.

Publication bias and small‐study effects were assessed through visual inspection of funnel plots and formally evaluated using Egger's linear regression test. Funnel plots were additionally examined following sensitivity analyses excluding smaller studies. Statistical analyses were performed using the R software version 4.3 (R Foundation for Statistical Computing, Vienna, Austria) with the ‘meta’ and ‘metafor’ packages. Only studies reporting sufficient raw data for RR calculation were included, and no conversions from odds ratios or other effect measures to RR were performed. A narrative synthesis was conducted to summarise factors and predictors associated with shock in children with MIS‐C, as quantitative meta‐analysis of predictors was not feasible because of heterogeneity in reported variables, outcome definitions and statistical reporting across studies.

## 3 Certainty of Evidence Assessment

### 3.1 Results

#### 3.1.1 Literature Search

**FIGURE 1:**
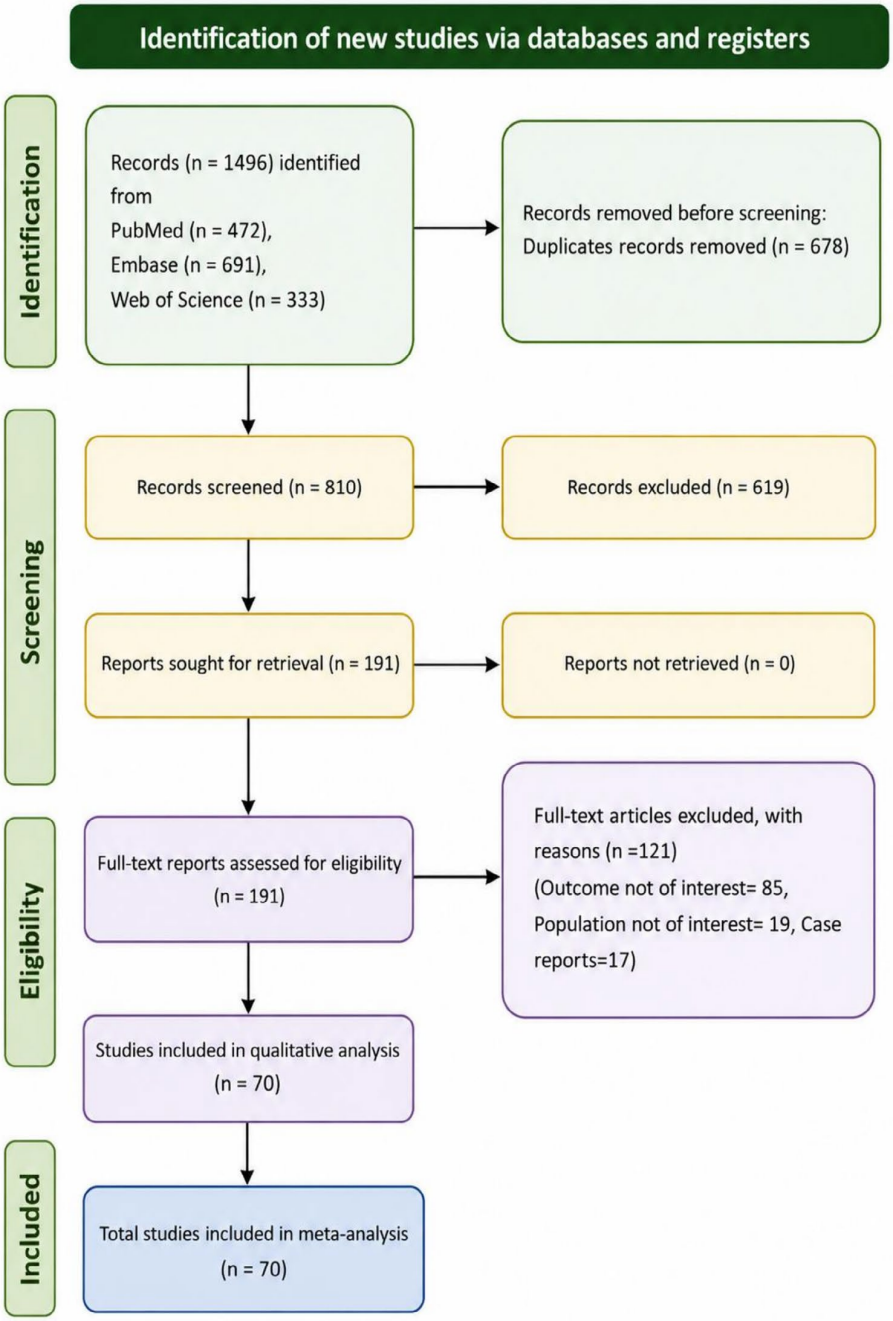
PRISMA flowchart depicting article selection and screening process.

The PRISMA flowchart in Figure 1 depicts the article selection and screening process. A total of 1496 records were found from various databases. After removing duplicates, 810 records were screened. Out of these, 619 records were excluded. After this screening process, 191 reports were retrieved for full‐text assessment. A further 121 studies were excluded. Reasons for exclusion include reports not being relevant to the topic of the study (e.g., outcomes or populations not of interest) or being case reports. Finally, 70 studies [2, 7, 13, 14, 15, 16, 17, 23, 24, 25, 26, 27, 28, 29, 30, 31, 32, 33, 34, 35, 36, 37, 38, 39, 40, 41, 42, 43, 44, 45, 46, 47, 48, 49, 50, 51, 52, 53, 54, 55, 56, 57, 58, 59, 60, 61, 62, 63, 64, 65, 66, 67, 68, 69, 70, 71, 72, 73, 74, 75, 76, 77, 78, 79, 80, 81, 82, 83, 84, 85] were included in the qualitative analysis, and the same number were included in the meta‐analysis.

#### 3.1.2 Characteristics of Included Studies

The characteristics of the included studies are given in Table 1. The analysis incorporated various observational studies investigating MIS‐C. These studies encompassed retrospective designs, where researchers analysed medical records after the fact; prospective designs, where they followed participants over time; and observational designs, where they collected data without manipulating any variables. Diagnoses were predominantly based on criteria set forth by the Centers for Disease Control and Prevention (CDC) or the World Health Organization (WHO). However, some studies adhered to guidelines from other organizations. The studies encompassed a substantial spectrum in sample size, with some enrolling a mere six participants and others exceeding 4000. Patient demographics also exhibited considerable heterogeneity. The median age ranged from 2 to 12 years old, underscoring the potential for MIS‐C to impact a broad age group within the paediatric population. Most studies reported a higher percentage of male patients compared to females. The quality assessment of the studies is presented in Table S3 for prevalence studies and Table S4 for risk assessment studies.

**TABLE 1:** Characteristics of included studies.

Study	Study design	Country	MIS‐C diagnosis criteria	Mean/median age (in years)	Total sample size	Male %	Total MIS‐C cases	No. of shock cases in MIS‐C
Abdelaziz 2023 [65]	Cross‐sectional study	Egypt	CDC	4 (median)	45	53.3	45	20
Abrams 2021 [64]	Retrospective study	USA	CDC	NA	1080	55.7	1080	392
Acevedo 2021 [70]	Observational cohort study	Colombia	WHO	7 (median)	78	59.0	78	68
Acharyya 2022 [25]	Retrospective study	India	RCPCH	10 (median)	17	35.3	17	5
Al Maskari 2021 [59]	Case series	Oman	NHS	8.3 (mean)	6	66.7	6	3
Alam 2023 [83]	Case series	India	WHO	6.5 (mean)	98	68.4	98	39
Almoosa 2020 [77]	Case series	Saudi Arabia	CDC	7 (median)	10	50.0	10	10
Angurana 2021 [34]	Retrospectively study	India	WHO	7 (median)	40	65.0	40	32
Avrusin 2023 [2]	Retrospective study	Russia	WHO	8.2 (mean)	166	59.6	166	72
Avrusin 2023 [67]	Retrospective study	Russia	WHO	8.2 (mean)	166	59.6	166	72
Avrusin 2024 [76]	Retrospective study	Russia	WHO & CDC	8.6 (mean)	79	NA	79	20
Awasthi 2022 [28]	Cross‐sectional	India	WHO	7 (median)	40	65.0	40	32
Bagri 2022 [39]	Prospective longitudinal study	India	WHO	8 (median)	31	61.3	31	18
Barrio 2023 [17]	Prospective study	Spain	RCPCH	8.9 (mean)	298	62.4	208	160
Bautista‐Rodriguez 2021 [30]	Retrospective study	Multinational	RCPCH	7 (median)	183	59.6	183	79
Belay 2021 [62]	Cross‐sectional study	USA	CDC	9 (median)	1733	57.4	1733	937
Belhadjer 2020 [33]	Retrospectively study	France	CDC	10 (median)	35	51.4	35	28
Benvenuto 2023 [71]	Retrospective study	Italy	WHO	12 (median)	20	75.0	20	11
Bichali 2023 [7]	Prospective cohort study	France	WHO	8.8 (mean)	28	57.1	11	11
Bichali 2024 [58]	Observational study	France	WHO	8.8 (mean)	37	43.0	34	14
Borgi 2021 [60]	Retrospective study	Tunisia	WHO	8 (median)	8	87.5	8	3
Campos 2023 [84]	Ambidirectional cohort study	Brazil	WHO/CDC	4.2 (mean)	112	59.8	112	32
Carbajal 2021 [55]	Retrospective study	France	WHO	7.5 (mean)	47	28.0	7	6
Caro‐Dominguez 2021 [15]	Retrospective study	Spain	RCPCH	8 (median)	37	56.8	37	1
Cattalini 2021 [69]	Retrospective study	Italy	AHA criteria	NA	149	NA	53	20
Chandran 2021 [50]	Retrospective study	India	WHO	5 (median)	17	6.0	17	16
Chattopadhyay 2022 [56]	Case series	India	WHO/CDC	4 (median)	17	64.7	17	13
Cheung 2020 [66]	Case series	USA	NA	8 (median)	17	47.1	17	13
Chinniah 2023 [14]	Retrospective study	South Africa	CDC	4.5 (mean)	29	44.8	29	17
Chiotos 2020 [74]	Case series	USA	CDC	8.5 (mean)	6	16.7	6	6
Davies 2020 [82]	Observational study	UK	RCPCH	11 (median)	78	66.7	78	68
Dhooria 2021 [72]	Cross‐sectional study	India	WHO	7.89 (mean)	34	82.4	34	15
Elilarasi 2022 [86]	Observational study	India	RCPCH	5.65 (mean)	65	45.0	65	27
Farias 2023 [49]	Prospective cohort study	Brazil	WHO	3.5 (mean)	208	56.3	67	28
García‐Domínguez 2023 [78]	Retrospective study	Mexico	WHO	6.5 (mean)	81	60.5	19	11
Garcia‐Salido 2020 [79]	Prospective study	Spain	RCPCH	8.1 (mean)	74	62.2	45	38
Grama 2022 [36]	Retrospective study	Romania	CDC & WHO	6.8 (mean)	34	61.8	34	19
Gupta Dch 2021 [63]	Retrospective cohort study	India	WHO	NA	41	63.4	20	13
Jain 2020 [44]	Observational study	India	WHO	7.2 (mean)	23	47.8	23	15
Jaxybayeva 2022 [32]	Retrospective study	Kazakhstan	CHC & WHO	6 (median)	89	71.9	89	15
Khoury 2023 [45]	Cohort study	USA	CDC	2.5 (mean)	1767	58.2	1429	1426
Kostik 2022 [51]	Retrospective cohort study	Russia	WHO & CDC	8.9 (mean)	122	56.6	122	51
Lampidi 2024 [54]	Retrospective study	Greece	WHO	8.2 (mean)	145	66.0	145	20
Lee 2023 [61]	Retrospective study	South Korea	CSTE and CDC	9.3 (mean)	31	35.5	31	6
Legarda 2023 [16]	Retrospective Cohort study	Ecuador	CDC	3 (median)	167	56.9	98	83
Lima‐Setta 2021 [53]	Prospective cohort study	Brazil	WHO	6.2 (mean)	56	69.6	56	33
Lopez 2022 [38]	NA	Canada	WHO	6 (median)	52	46.6	11	10
Maggio 2023 [73]	Cohort study	Italy	CDC & WHO	8.3 (mean)	22	50.0	22	2
Maheshwari 2022 [81]	Prospective study	India	WHO	3 (median)	29	48.3	29	15
Mahmoud 2021 [57]	Retrospective study	Egypt	NA	7 (median)	64	59.4	64	45
Mannarino 2022 [37]	Retrospective study	Italy	CDC	10 (median)	32	75.0	10	8
Mehra 2021 [24]	Retrospective cohort Study	India	CDC	7 (median)	120	70.0	120	73
Menchaca‐Aguayo 2022 [47]	Cross‐sectional study	Mexico	WHO/CDC	7.5 (mean)	90	47.8	90	41
Miller 2022 [27]	Cross‐sectional study	USA	CDC	9 (median)	4470	59.8	4470	2018
Nadua 2022 [13]	Observational study	Singapore	Singapore Ministry of Health criteria	7.5 (mean)	12	66.7	12	6
Nayak 2022 [52]	Retrospective study	India	WHO	7 (median)	134	67.2	134	47
Nino‐Taravilla 2021 [29]	Observational study	Chile	Ministry of Public Health of Chile	6.5 (mean)	26	NA	26	24
Pawar 2021 [43]	Case series	India	CDC	2 (median)	20	50.0	20	5
Rekhtman 2021 [68]	Cohort study	USA	AHA criteria	10.5 (mean)	31	61.3	19	11
Renson 2023 [40]	Cohort study	Canada	WHO	6 (median)	57	71.9	57	50
Sen 2023 [75]	Prospective study	India	WHO	4.8 (mean)	35	60.0	35	20
Shahbaznejad 2020 [80]	Case series	Iran	WHO	5.37 (mean)	10	60.0	10	2
Shobhavat 2020 [85]	Case series	India	WHO	7 (median)	21	47.6	20	20
Sperotto 2023 [46]	Retrospective study	Multinational	CDC, RCPCH, or WHO	9.7 (mean)	598	61.0	598	84
Toubiana 2021 [48]	Retrospective study	France	AHA criteria	NA	23	NA	23	14
Valverde 2021 [31]	Retrospective study	Europe	CDC	8.4 (mean)	286	67.8	286	115
Varadarajan 2023 [26]	Prospective study	India	WHO	6 (median)	257	NA	257	118
Varga 2023 [42]	Ambidirectional cohort study	Hungary	CDC	7 (median)	53	60.4	53	17
Whittaker 2020 [23]	Case series	United Kingdom	CDC and WHO	9 (median)	58	65.5	58	29
Yilmaz Ciftdogan 2022 [41]	Retrospective study	Turkey	CDC & WHO	7 (median)	101	50.5	101	39

Abbreviations: AHA, American Heart Association; CDC, Centers for Disease Control and Prevention; CHC, Children's Hospital Colorado; CSTE, Council of State and Territorial Epidemiologists; MIS‐C, Multisystem inflammatory syndrome in children; NHS, National Health Service; RCPCH, Royal College of Paediatrics and Child Health; WHO, World Health Organization.

#### 3.1.3 Incidence of Shock Among Children With MIS‐C

**FIGURE 2:**
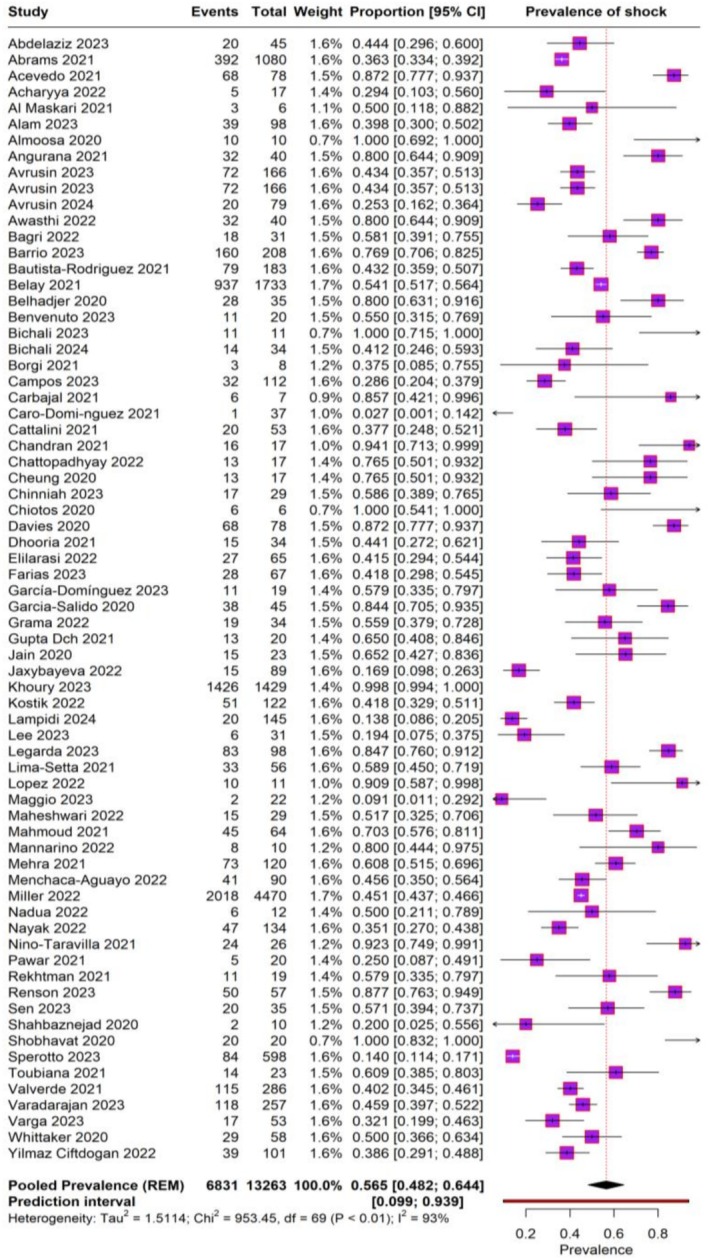
Prevalence of shock among children with MIS‐C.

The results of the meta‐analysis investigating the incidence of shock in children with MIS‐C reveal a pooled prevalence from the included studies (Figure 2). The meta‐analysis, with data from a diverse range of studies, indicated a pooled prevalence of shock at 56.5% (95% CI: 0.482–0.644). This result suggests that, on average, more than half of the children with MIS‐C experienced shock, reflecting a considerable clinical burden. The heterogeneity of the analysis, as indicated by the *I* ^2^ statistic (*I* ^2^ = 95.45%), points to a high degree of variability in the incidence rates reported across the studies. The prediction interval was found to be 0.099–0.93.

### 3.2 Sensitivity Analysis

A sensitivity analysis was conducted by excluding studies with a sample size of fewer than 50 participants to assess the robustness of the pooled prevalence estimate of shock among children with MIS‐C (Figure S1). After exclusion of smaller studies, the pooled prevalence of shock remained high at 50% (95% CI: 40%–59%), which was comparable to the primary analysis, indicating that the overall findings were stable and not disproportionately influenced by small studies. Although substantial heterogeneity persisted across studies (*I* ^2^ = 99.2%, *p* < 0.001), the consistency of the pooled prevalence estimate supports the reliability of the association between MIS‐C and shock. The prediction interval remained wide (0.06–0.94), reflecting considerable between‐study variability in shock prevalence across different study populations and clinical settings.

An additional sensitivity analysis was performed by excluding studies with a sample size of fewer than 100 participants to further evaluate the robustness of the pooled prevalence estimate of shock among children with MIS‐C (Figure S2). Following exclusion of smaller studies, the pooled prevalence of shock was 46% (95% CI: 33%–59%), which remained consistent with the primary analysis and the previous sensitivity analysis excluding studies with fewer than 50 participants. These findings suggest that the overall prevalence estimate was stable and not substantially affected by smaller studies. Despite persistent high heterogeneity among the included studies (*I* ^2^ = 99.6%, *p* < 0.001), the direction and magnitude of the pooled estimate remained largely unchanged. The wide prediction interval (0.03–0.93) indicates continued variability in shock prevalence across different study settings and populations. Overall, the sensitivity analysis confirmed the robustness and reliability of the pooled prevalence findings.

### 3.3 Subgroup Analysis by Country

Subgroup analysis based on year of publication demonstrated variation in the pooled prevalence of shock among children with MIS‐C across different time periods (Figure S3). Studies published in 2020 showed the highest pooled prevalence of shock at 80% (95% CI: 60%–95%; *I* ^2^ = 85.3%), followed by studies published in 2021 with a pooled prevalence of 55% (95% CI: 44%–68%; *I* ^2^ = 93.4%). Studies published in 2022 and 2023 demonstrated comparable prevalence estimates of 50% (95% CI: 39%–61%; *I* ^2^ = 83.5%) and 54% (95% CI: 39%–68%; *I* ^2^ = 99.4%), respectively. In contrast, studies published in 2024 showed a lower pooled prevalence of shock at 25% (95% CI: 1%–63%; *I* ^2^ = 84.3%), although this subgroup included fewer studies and participants.

Overall, the pooled prevalence of shock across all studies was 56% (95% CI: 49%–63%), with substantial heterogeneity observed between studies (*I* ^2^ = 98.4%). A statistically significant difference was identified between publication‐year subgroups (test for subgroup differences: *χ* ^2^ = 20.06, df = 4, *p* = 0.0005), suggesting a temporal decline in the prevalence of shock among children with MIS‐C over the course of the pandemic.

### 3.4 Subgroup Analysis by Diagnostic Criteria

Subgroup analysis according to the diagnostic criteria used for MIS‐C demonstrated variability in the pooled prevalence of shock across different case definitions (Figure S4). Studies utilizing the CDC criteria reported the highest pooled prevalence among the major subgroups at 65% (95% CI: 47%–81%; *I* ^2^ = 99.5%). Studies using WHO criteria demonstrated a pooled prevalence of 61% (95% CI: 50%–71%; *I* ^2^ = 92.1%), while studies applying RCPCH criteria reported a pooled prevalence of 52% (95% CI: 20%–83%; *I* ^2^ = 96.6%).

Among studies using combined diagnostic definitions, the pooled prevalence was 38% (95% CI: 20%–57%; *I* ^2^ = 83.0%) for studies applying WHO or CDC criteria, and 47% (95% CI: 0%–100%; *I* ^2^ = 51.9%) for studies using combined CDC and WHO criteria. Studies based on AHA criteria demonstrated a pooled prevalence of 50% (95% CI: 19%–81%; *I* ^2^ = 54.2%), whereas studies with unspecified diagnostic criteria reported a pooled prevalence of 72% (95% CI: 42%–94%; *I* ^2^ = 0%).

Several additional diagnostic frameworks were represented by single studies, including CHC and WHO criteria, CSTE and CDC criteria, Singapore Ministry of Health criteria, Ministry of Public Health of Chile criteria and combined CDC/RCPCH/WHO criteria, with prevalence estimates ranging from 14% to 92%.

Overall, the pooled prevalence of shock across all studies was 56% (95% CI: 49%–63%), with substantial heterogeneity observed (*I* ^2^ = 98.4%). A statistically significant difference was identified between diagnostic‐criteria subgroups (test for subgroup differences: *χ* ^2^ = 499.91, df = 12, *p* < 0.0001), suggesting that variations in MIS‐C diagnostic definitions may partly contribute to differences in reported shock prevalence across studies.

### 3.5 Subgroup Analysis by Year

Subgroup analysis according to year demonstrated temporal variation in the pooled prevalence of shock among children with MIS‐C (Figure S5). Studies published in 2020 reported the highest pooled prevalence of shock at 80% (95% CI: 60%–95%; *I* ^2^ = 85.3%). In comparison, studies published in 2021 demonstrated a pooled prevalence of 55% (95% CI: 44%–68%; *I* ^2^ = 93.4%), while studies published in 2022 and 2023 reported prevalence estimates of 50% (95% CI: 39%–61%; *I* ^2^ = 83.5%) and 54% (95% CI: 39%–68%; *I* ^2^ = 99.4%), respectively. Studies published in 2024 showed the lowest pooled prevalence at 25% (95% CI: 1%–63%; *I* ^2^ = 84.3%), although this subgroup included relatively few studies and participants.

The overall pooled prevalence of shock across all included studies was 56% (95% CI: 49%–63%), with substantial heterogeneity observed (*I* ^2^ = 98.4%). A statistically significant difference was identified between publication‐year subgroups (test for subgroup differences: *χ* ^2^ = 20.06, df = 4, *p* = 0.0005), suggesting a possible decline in the prevalence of shock among children with MIS‐C over the course of the COVID‐19 pandemic.

### 3.6 Meta‐Regression

Univariable and multivariable meta‐regression analyses were conducted to investigate potential sources of heterogeneity in the pooled prevalence of shock among children with MIS‐C (Table S5). In univariable analysis, publication year was significantly associated with shock prevalence (*β* = −0.0797, *p* = 0.0072), suggesting a decreasing prevalence of shock over time. However, this association was attenuated and no longer statistically significant in the multivariable model (*β* = −0.0671, *p* = 0.0625). Mean/median age, male proportion, sample size and diagnostic criteria were not significantly associated with shock prevalence in either univariable or multivariable analyses. The multivariable model explained 6.3% of the between‐study heterogeneity, although substantial residual heterogeneity remained (*I* ^2^ = 95.63%).

### 3.7 Risk of Shock in MIS‐C

To address heterogeneity arising from differences in comparator populations, analyses were performed according to comparator type. Studies comparing MIS‐C with Kawasaki disease were analysed separately from studies comparing MIS‐C with acute/severe COVID‐19.

**FIGURE 3:**
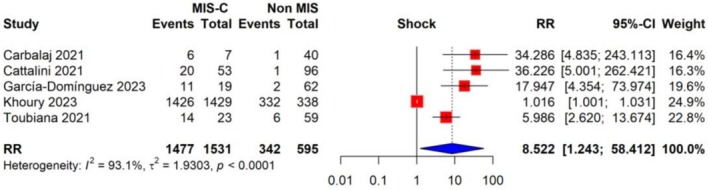
Forest plot showing the pooled RR of shock in children with multisystem inflammatory syndrome in children (MIS‐C) compared with children with Kawasaki disease only without MIS‐C.

**FIGURE 4:**
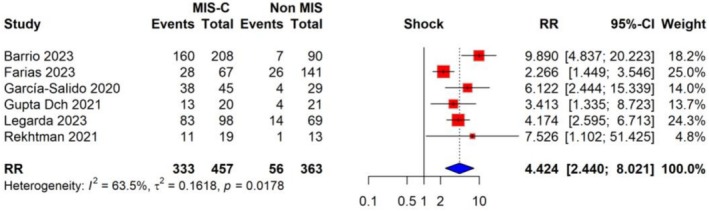
Forest plot showing the pooled RR of shock in children with multisystem inflammatory syndrome in children (MIS‐C) compared with children with acute/severe COVID‐19 without MIS‐C.

In the analysis comparing MIS‐C with Kawasaki disease (Figure 3), children with MIS‐C demonstrated a significantly higher risk of shock. The pooled RR was 8.522 (95% CI: 1.243–58.412), indicating that shock occurred more frequently in children with MIS‐C than in those with Kawasaki disease. Although the association was statistically significant:

Similarly, in the meta‐analysis comparing MIS‐C with acute/severe COVID‐19 (Figure 4), children with MIS‐C had a significantly increased risk of shock compared to children with non‐MIS‐C COVID‐19. The pooled RR was 4.424 (95% CI: 2.440–8.021), corresponding to an approximately fourfold increased risk of shock in MIS‐C. Moderate heterogeneity was observed across studies (*I* ^2^ = 63.5%, *p* = 0.0178).

### 3.8 Factors and Predictors of Shock in MIS‐C

A narrative synthesis of the included studies was performed to summarize reported clinical, echocardiographic and laboratory predictors associated with shock among children with MIS‐C. Several studies have pinpointed crucial echocardiographic and laboratory parameters in exploring factors of shock in MIS‐C. Varadarajan et al. [26] identified significant associations between shock and echocardiographic features such as mitral regurgitation (*p* = 0.029), hyperechogenic coronaries (*p* = 0.006), left ventricular dysfunction (*p* < 0.001) and low ejection fraction (*p* = 0.007). Likewise, myocardial dysfunction or reduced left ventricular ejection fraction was observed by Angurana et al. (*p* = 0.02) and Mannarino et al. [37], who specifically pointed to LVEF < 45% (*p* < 0.01) as being associated with shock.

From a demographic and clinical viewpoint, Chinniah et al. [14] reported that the female sex had an odds ratio (OR) of 9.75 (1.74–54.85) for the development of shock. They also found significant associations with cardiac involvement (OR = 7.20, 95% CI: 1.35–38.33), altered level of consciousness (ALOC) (OR = 11.37, 95% CI: 1.17–590.4), and various laboratory markers such as platelets, albumin, C‐reactive protein (CRP), D‐dimer, increased serum ferritin, B‐type natriuretic peptide (BNP), troponin and creatinine. Jain et al. [44] further highlighted the role of hematologic and biochemical markers, identifying increased neutrophil percentages (*p* = 0.007), decreased lymphocyte percentage (*p* = 0.02), decreased platelets (*p* = 0.06), increased serum glutamic pyruvic transaminase (*p* = 0.007), increased serum ferritin (*p* = 0.01), increased NT‐proBNP (*p* = 0.05) and raised troponin levels (*p* = 0.007) as being significantly associated with shock.

The strong association between acute kidney injury (AKI) and shock was reported by Lampidi et al. [54] (OR = 8.537, 95% CI: 2.088–34.905, *p* = 0.003) and Chinniah et al. [14] (OR = 20.17, 95% CI: 2.07–196.4), highlighting AKI as a critical predictor of shock in MIS‐C.

### 3.9 Publication Bias

**FIGURE 5:**
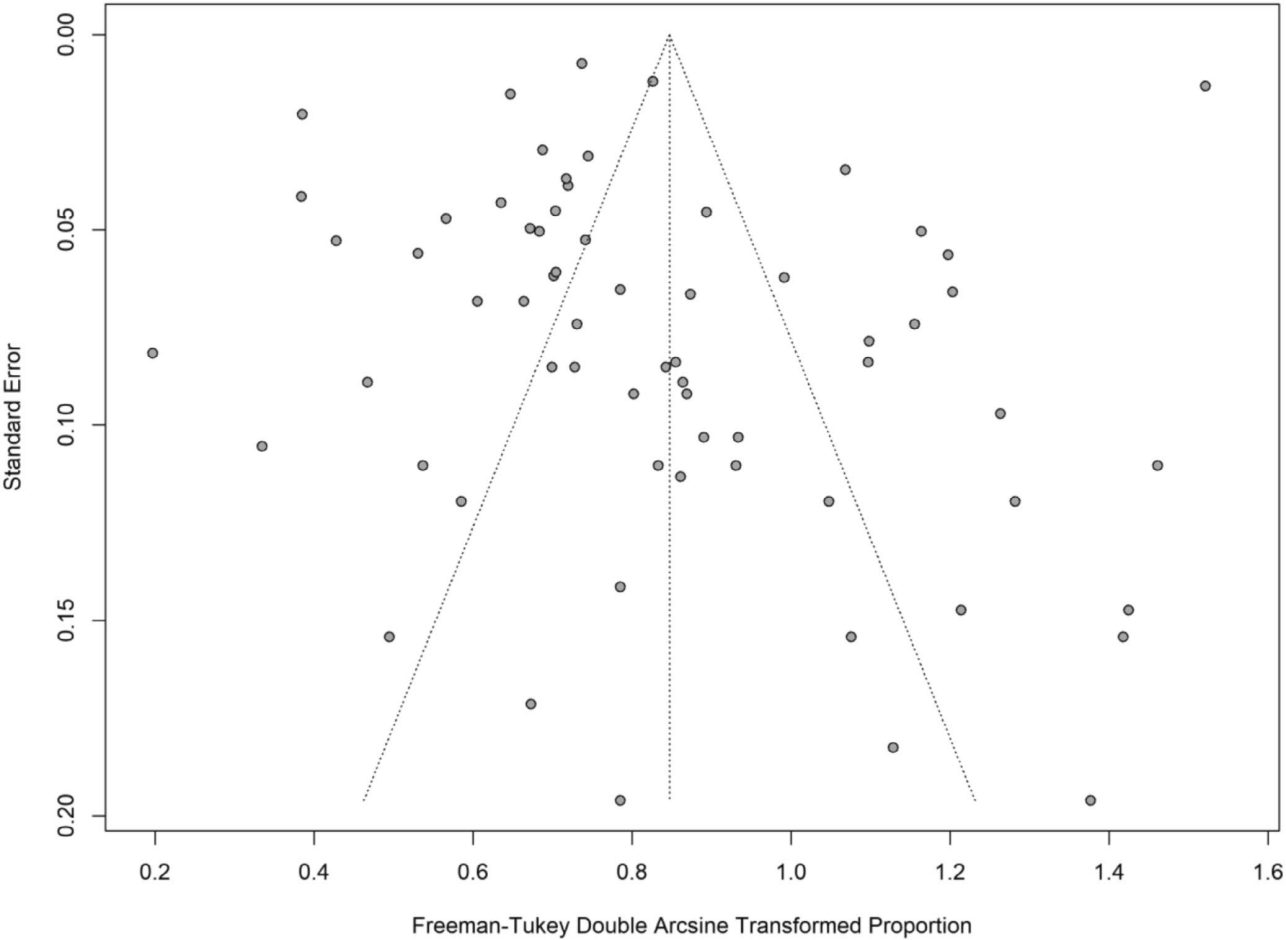
Funnel plot assessing publication bias in the prevalence of shock among children with MIS‐C.

Publication bias was assessed through visual inspection of funnel plots and Egger's linear regression test. The primary funnel plot for the pooled prevalence of shock among children with MIS‐C demonstrated mild asymmetry, with several smaller studies dispersed outside the pseudo 95% confidence limits (Figure 5). The wider spread among studies with larger standard errors suggested substantial between‐study heterogeneity. However, the overall distribution remained relatively balanced around the pooled estimate without a clear directional pattern indicating major publication bias.

Egger's regression test showed no statistically significant evidence of funnel plot asymmetry or small‐study effects in the primary analysis (*t* = 0.03, df = 68, *p* = 0.9784). The estimated bias coefficient was 0.0359 (SE = 1.3202), indicating minimal asymmetry.

Sensitivity analyses were additionally performed after excluding studies with sample sizes fewer than 50 and fewer than 100 participants. Funnel plots from both sensitivity analyses continued to show mild asymmetry and study dispersion, likely reflecting residual clinical and methodological heterogeneity rather than true publication bias (Figures S6 and S7). Egger's regression test remained non‐significant after exclusion of studies with sample size < 50 (*t* = −0.47, df = 30, *p* = 0.6386) and after exclusion of studies with sample size < 100 (*t* = −0.58, df = 15, *p* = 0.5682). These findings suggest that publication bias was unlikely to have substantially influenced the pooled prevalence estimates, and that the overall results remained robust following restriction to larger studies.

### 3.10 GRADE Certainty of Evidence

Using the GRADE framework, the certainty of evidence for the pooled prevalence of shock among children with MIS‐C was rated as very low because of substantial heterogeneity and methodological variability across included studies (Table S6). The certainty of evidence for the comparative risk of shock between MIS‐C and Kawasaki disease cohorts was also rated as very low owing to risk of bias and imprecision. In contrast, the certainty of evidence for the increased risk of shock in MIS‐C compared with acute/severe COVID‐19 cohorts was rated as low. Evidence regarding predictors of shock was considered very low certainty because findings were derived from heterogeneous observational studies synthesized narratively without quantitative pooling.

## 4 Discussion

The systematic review and meta‐analysis presented here aim to shed light on the prevalence, relative risk, and predictors of shock in children affected by MIS‐C in the context of the COVID‐19 pandemic. This condition, which manifests as a hyperinflammatory response to SARS‐CoV‐2 infection, presents a considerable challenge in paediatric healthcare due to its potential to rapidly progress to severe complications, including shock. Our comprehensive analysis, based on a pool of 70 studies, reveals significant insights into the burden of shock in MIS‐C, its associated risk factors, and the potential pathways for early identification and intervention.

Our findings indicate a pooled prevalence of shock in MIS‐C patients at 56.5%, suggesting that more than half of the children with MIS‐C may experience this life‐threatening complication. This high prevalence indicates the critical nature of shock in the context of MIS‐C and highlights the need for vigilant monitoring and management of patients diagnosed with this syndrome. The analysis further elucidates that children with MIS‐C have an increased risk of developing shock compared to those without MIS‐C. This significantly elevated risk confirms the severe impact of MIS‐C on the cardiovascular system and its role in precipitating shock.

Our review identified several predictors of shock in children with MIS‐C. Echocardiographic abnormalities such as mitral regurgitation, hyperechogenic coronaries, left ventricular dysfunction and reduced ejection fraction were significantly associated with shock, pointing to the central role of myocardial involvement in the pathophysiology of MIS‐C‐related shock. Demographic factors such as gender and age were unclear in the studies. Clinical features including cardiac involvement and altered levels of consciousness were also identified as significant predictors. Laboratory markers, including elevated levels of C‐reactive protein, D‐dimer, serum ferritin, B‐type natriuretic peptide, troponin and creatinine, emerged as critical predictors, reflecting the systemic inflammatory response and organ dysfunction in MIS‐C. The strong association between AKI and shock further highlights the multi‐organ impact of this syndrome and the interconnectedness of organ systems in determining the severity of the patient's condition.

Previous systematic reviews have explored the clinical manifestation of MIS‐C. For instance, the study by Santos et al. [87] found that fever was universally present (100%) in cases of MIS‐C, while gastrointestinal symptoms were observed in 82% of cases, and abdominal pain was noted in 68% of individuals. Manifestations of shock or hypotension were frequently encountered among MIS‐C patients. Symptoms related to the cardiac system (66%) were more prevalent compared to those affecting the respiratory (39%) or neurological systems (28%) [87]. Another systematic review reported that MIS‐C typically targets children between 6 and 12 years, with a higher frequency in boys, affecting various systems including the gastrointestinal, cardiovascular and respiratory systems [88]. Radiographic and laboratory findings indicate systemic inflammation, with elevated inflammatory markers, neutrophil activation and signs of organ involvement. Distinguishing MIS‐C from COVID‐19 relies on specific inflammatory markers and autoantibody profiles. Treatment mainly consists of immunomodulatory therapies like IVIG and glucocorticoids, with ECMO and mechanical ventilation reserved for severe cases. Most children recover within a week of hospital discharge [88].

Recent evidence has also identified several factors associated with the development of MIS‐C itself following SARS‐CoV‐2 infection. A systematic review and meta‐analysis by Rayner et al. [89] reported that younger age (< 12 years), male sex and Black race were probably associated with an increased risk of developing MIS‐C, whereas malignancy and underlying respiratory disease were associated with a lower risk. The authors also noted possible associations with Asian race and lower prevalence of comorbidities. These findings suggest that demographic and host‐related factors may influence susceptibility to MIS‐C and may partially contribute to the variability in disease severity and shock prevalence observed across studies included in the present review [90].

Given the high prevalence of shock in this patient population, clinicians must be alert to the early signs of cardiovascular compromise [91]. The identification of significant predictors of shock, including echocardiographic abnormalities, laboratory markers indicative of inflammation and organ dysfunction and clinical features such as altered levels of consciousness, necessitates the integration of these parameters into routine assessment and monitoring protocols. This approach will enable the early detection of children at heightened risk of developing shock, facilitating timely and aggressive interventions that may include fluid resuscitation, inotropic support and immunomodulatory therapies [92, 93]. Furthermore, the development of predictive models based on the identified risk factors could significantly enhance risk stratification, inform clinical decision‐making and optimize resource allocation. By understanding and applying these findings, clinicians can improve the outcomes of children with MIS‐C through early identification, risk stratification and targeted therapeutic strategies, thereby mitigating the progression to shock and reducing the associated morbidity and mortality.

The development of prognostic models incorporating these risk factors could aid in risk stratification and personalized management of MIS‐C patients, optimizing healthcare resources and outcomes. Future research should focus on unravelling the underlying mechanisms of MIS‐C and its progression to shock, exploring the genetic and environmental factors that contribute to variability in disease severity and response to treatment. Longitudinal studies are needed to assess the long‐term outcomes of children with MIS‐C who experience shock, including the potential for chronic cardiovascular sequelae. Additionally, clinical trials evaluating the efficacy of interventions targeting the identified risk factors could provide evidence‐based guidelines for the management of MIS‐C.

This study is not without limitations. The significant heterogeneity among the included studies indicates that the findings should be interpreted with caution. The prevalence and risk estimates may be influenced by the overrepresentation of studies with higher effect sizes, necessitating careful consideration of the overall effect size estimates. This issue, coupled with the limited reporting on the factors affecting the risk of shock and the exclusion of non‐English language studies or research outside selected databases, might have led to an incomplete synthesis of global evidence. The reliance on retrospective studies limits the ability to draw causal inferences between identified predictors and the development of shock, highlighting associations that may be influenced by uncontrolled confounding factors. Together, these limitations emphasize the necessity for further research, employing diverse and rigorous methodologies, to refine our understanding of MIS‐C and optimize the management of its severe complications. The substantial heterogeneity and wide prediction intervals observed across studies indicate considerable variability in study populations, diagnostic criteria and clinical management strategies. Therefore, additional studies alone may not sufficiently improve the current evidence base. Future research should focus on large, high‐quality prospective multicenter studies using standardized MIS‐C definitions, uniform shock criteria and consistent reporting of clinical and laboratory predictors to improve the precision and applicability of findings.

## 5 Conclusion

Our analysis revealed significant burden and risk for shock in children with MIS‐C, with a pooled prevalence indicating that more than half of affected children may experience this complication. The findings underscore the importance of early identification of children at high risk for shock, through vigilant monitoring for echocardiographic abnormalities, demographic and clinical predictors, and elevated laboratory markers indicative of systemic inflammation and organ dysfunction. Further large, methodologically robust prospective studies with standardized diagnostic and outcome definitions are required to better characterize the predictors, risk factors and outcomes of shock in children with MIS‐C.

## Funding

The authors have nothing to report.

## Ethics Statement

Ethics approval was not required for the study as it is a secondary analysis of existing data that was previously published.

## Consent

The authors have nothing to report.

## Conflicts of Interest

The authors declare no conflicts of interest.

## Supporting information


**Table S1:** PRISMA Checklist.
**Table S2:** Search strategy.
**Table S3:** Quality assessment of studies with the JBI tool.
**Table S4:** Quality assessment of studies with the ROBINS‐E for risk assessment.
**Table S5:** Meta‐regression result.
**Table S6:** GRADE assessment for prevalence and comparative risk outcomes.
**Figure S1:** Sensitivity analysis after excluding using studies less than 50 sample size.
**Figure S2:** Sensitivity analysis after excluding using studies less than 100 sample size.
**Figure S3:** Subgroup analysis by country.
**Figure S4:** Subgroup analysis by diagnostic criteria.
**Figure S5:** Subgroup analysis by year.
**Figure S6:** Funnel plot after excluding studies less than 50 sample size.
**Figure S7:** Funnel plot after excluding studies less than 100 sample size.

## Data Availability

The data that support the findings of this study are available from the corresponding author upon reasonable request.
