# Supplementary material for: Prevalence, Comparative Risk, and Predictors of Shock in Children With Multisystem Inflammatory Syndrome Associated With COVID‐19: A Systematic Review and Meta‐Analysis
Source: J Paediatr Child Health. 2026 Jun 10;62(8):1346–62. doi: 10.1111/jpc.70470 (PMC13480730; doi:10.1111/jpc.70470)
Supplement: Supplementary file 1 [file JPC-62-1346-s001.docx]

**Supplementary Materials**

**Table S1.** PRISMA Checklist

**Table S2.** Search strategy

**Table S3.** Quality assessment of studies with the JBI tool

**Table S4.** Quality assessment of studies with the ROBINS-E for risk assessment

**Table S5:** Meta-regression result

## **Table S1.** PRISMA Checklist

| **Section and Topic** | **Item #** | **Checklist Item** | **Reported on Page #** | **Approximate Line #** |
| --- | --- | --- | --- | --- |
| TITLE | 1 | Identify the report as a systematic review. | Page 1 | Title section |
| ABSTRACT | 2 | See the PRISMA 2020 for Abstracts checklist. | Page 1 | Abstract lines 1–32 |
| INTRODUCTION | 3 | Describe the rationale for the review in the context of existing knowledge. | Pages 1–3 | Introduction lines 1–65 |
| INTRODUCTION | 4 | Provide an explicit statement of the objective(s) or question(s) the review addresses. | Page 3 | Introduction final paragraph |
| METHODS | 5 | Specify the inclusion and exclusion criteria for the review and how studies were grouped for the syntheses. | Pages 4–5 | Inclusion and Exclusion Criteria section |
| METHODS | 6 | Specify all databases, registers, websites, organisations, reference lists and other sources searched or consulted. | Page 4 | Literature Search section |
| METHODS | 7 | Present the full search strategies for all databases, registers and websites. | Supplementary Table S2 | NA |
| METHODS | 8 | Specify the methods used to decide whether a study met the inclusion criteria. | Pages 5–6 | Selection of Studies section |
| METHODS | 9 | Specify the methods used to collect data from reports. | Page 6 | Data Extraction and Quality Assessment section |
| METHODS | 10a | List and define all outcomes for which data were sought. | Pages 5–7 | Data extraction and statistical analysis sections |
| METHODS | 10b | List and define all other variables for which data were sought. | Page 6 | Data Extraction section |
| METHODS | 11 | Specify the methods used to assess risk of bias in the included studies. | Page 6 | Quality assessment paragraph |
| METHODS | 12 | Specify for each outcome the effect measure(s) used in the synthesis. | Pages 6–7 | Statistical Analysis section |
| METHODS | 13a | Describe the processes used to decide which studies were eligible for each synthesis. | Pages 5–7 | Selection and synthesis sections |
| METHODS | 13b | Describe any methods required to prepare the data for presentation or synthesis. | Pages 6–7 | Statistical Analysis section |
| METHODS | 13c | Describe methods used to tabulate or visually display results. | Pages 7–8 | Statistical Analysis section |
| METHODS | 13d | Describe methods used to synthesize results and provide rationale. | Pages 6–8 | Statistical Analysis section |
| METHODS | 13e | Describe any methods used to explore possible causes of heterogeneity. | Pages 7–8 | Subgroup and Meta-regression sections |
| METHODS | 13f | Describe any sensitivity analyses conducted. | Pages 7–8 | Sensitivity Analysis section |
| METHODS | 14 | Describe methods used to assess risk of bias due to missing results. | Page 8 | Publication Bias section |
| METHODS | 15 | Describe any methods used to assess certainty in the body of evidence. | Page 7 | Certainty of Evidence Assessment section |
| RESULTS | 16a | Describe the results of the search and selection process. | Pages 7–8 | Literature Search section and Figure 1 |
| RESULTS | 16b | Cite studies that appeared to meet inclusion criteria but were excluded. | Figure 1 / Supplement | Figure 1 |
| RESULTS | 17 | Cite each included study and present its characteristics. | Pages 8–14 | Table 1 |
| RESULTS | 18 | Present assessments of risk of bias for each included study. | Page 8 | Reference to Tables S3 and S4 |
| RESULTS | 19 | Present results of all statistical syntheses conducted. | Pages 8–13 | Results section |
| RESULTS | 20a | Summarise characteristics and risk of bias among contributing studies. | Pages 8–13 | Results section |
| RESULTS | 20b | Present results of all statistical syntheses. | Pages 8–13 | Figures 2–5 |
| RESULTS | 20c | Present results of investigations of heterogeneity. | Pages 9–11 | Subgroup and Meta-regression sections |
| RESULTS | 20d | Present results of all sensitivity analyses conducted. | Pages 8–9 | Sensitivity Analysis section |
| RESULTS | 21 | Present assessments of risk of bias due to missing results. | Page 12 | Publication Bias section |
| RESULTS | 22 | Present assessments of certainty in the body of evidence. | Page 12 | GRADE certainty of evidence section |
| DISCUSSION | 23a | Provide a general interpretation of the results. | Pages 12–15 | Discussion section |
| DISCUSSION | 23b | Discuss limitations of the evidence included in the review. | Pages 14–15 | Discussion limitations paragraph |
| DISCUSSION | 23c | Discuss limitations of the review processes used. | Pages 14–15 | Discussion limitations paragraph |
| DISCUSSION | 23d | Discuss implications of the results for practice, policy and future research. | Pages 13–15 | Discussion and Conclusion |
| OTHER INFORMATION | 24a | Provide registration information for the review. | Not reported | NA |
| OTHER INFORMATION | 24b | Indicate where the review protocol can be accessed. | Not reported | NA |
| OTHER INFORMATION | 24c | Describe and explain any amendments to protocol. | Not reported | NA |
| OTHER INFORMATION | 25 | Describe sources of financial or non-financial support. | Page 15 | Funding Declaration section |
| OTHER INFORMATION | 26 | Declare any competing interests of review authors. | Page 15 | Competing Interest section |
| OTHER INFORMATION | 27 | Report availability of data, code and other materials. | Page 15 | Availability of Data and Materials section |

**Notes**

- Exact line numbers may vary depending on journal formatting and typesetting.
- Supplementary materials include Tables S2–S6 and Figures S1–S7.
- It is recommended to enable continuous line numbering in the manuscript before submission to ensure precise reviewer referencing.

**Table S2. The adjusted search terms as per searched electronic databases**

| Database | No | Search Query | Results |
| --- | --- | --- | --- |
|  | |  | |
| PubMed | **#1** | **(child[Title/Abstract] OR children[Title/Abstract] OR "pediatric*"[Title/Abstract] OR "paediatric*"[Title/Abstract] OR "adolescent*"[Title/Abstract] OR "young adult"[Title/Abstract] OR "teen*"[Title/Abstract] OR infant[Title/Abstract] OR infants[Title/Abstract] OR newborn[Title/Abstract] OR newborns[Title/Abstract] OR neonate[Title/Abstract] OR neonates[Title/Abstract] OR juvenile[Title/Abstract] OR youth[Title/Abstract] OR toddler[Title/Abstract] OR baby[Title/Abstract] OR preadolescent[Title/Abstract])** | 2576256 |
|  | **#2** | **("SARS-CoV-2"[Title/Abstract] OR Coronavirus[Title/Abstract] OR "COVID-19"[Title/Abstract] OR "severe acute respiratory syndrome" Coronavirus[Title/Abstract] OR 2019-nCoV[Title/Abstract] OR "COVID*"[Title/Abstract])** | 399076 |
|  | **#3** | **(MIS-C[Title/Abstract] OR "Multisystem inflammatory syndrome"[Title/Abstract] OR "hyperinflammatory syndrome"[Title/Abstract] OR "inflammatory multisystem syndrome"[Title/Abstract] OR "multisystem inflammatory syndrome"[Title/Abstract] OR "Kawasaki‐like disease"[Title/Abstract] OR "Kawasaki Disease"[Title/Abstract] OR "hyperinflammatory syndrome"[Title/Abstract] OR "Multisystem inflammat*"[Title/Abstract])** | 11648 |
|  | **#4** | **("Circulatory Failure"[Title/Abstract] OR "Circulatory Collapse"[Title/Abstract] OR shock[Title/Abstract])** | 220251 |
|  | **#5** | **#1 AND #2AND#3AND#4** |  |
| EMBASE | **#1** | **child**:ab,ti OR **children**:ab,ti OR **adolescent**:ab,ti OR **'young adult'**:ab,ti OR **infant**:ab,ti OR **teen***:ab,ti OR **paediatric***:ab,ti OR **pediatric**:ab,ti OR **infants**:ab,ti OR **newborn**:ab,ti OR **newborns**:ab,ti OR **neonate**:ab,ti OR **neonates**:ab,ti OR **baby**:ab,ti OR **toddler**:ab,ti OR **preadolescent**:ab,ti OR **juvenile**:ab,ti OR **youth**:ab,ti | 3218778 |
|  | **#2** | **'sars-cov-2'**:ab,ti OR **'coronavirus'**:ab,ti OR **'covid-19'**:ab,ti OR **'severe acute respiratory syndrome coronavirus'**:ab,ti OR **'covid'**:ab,ti OR **'2019-n cov'**:ab,ti | 464766 |
|  | **#3** | **'mis-c'**:ab,ti OR **'hyperinflammatory syndrome'**:ab,ti OR **'inflammatory multisystem syndrome'**:ab,ti OR **'multisystem inflammatory syndrome'**:ab,ti OR **'kawasaki like disease'**:ab,ti OR **'kawasaki disease'**:ab,ti | 15174 |
|  | **#4** | **circulatory failure'**:ab,ti OR **'circulatory collapse'**:ab,ti OR **'shock'**:ab,ti | 287080 |
|  | **#5** | #1 AND #2AND #3 AND #4 | 706 |
| WOS  advanced | **#1** | (((((((((((((((((((((((((((TI=(Child)) OR TI=(Children)) OR TI=(Young adult)) OR TI=(Pediatric)) OR TI=(paediatric)) OR AB=(Child)) OR AB=(Pediatric)) OR AB=(children)) OR AB=(Young adult)) OR AB=(paediatric)) OR TI=(adolescent)) OR TI=(teen*)) OR AB=(adolescent)) OR AB=(teen*)) OR TI=(infant)) OR AB=(infant)) OR TI=(neonate)) OR AB=(neonates)) OR TI=(baby)) OR AB=(baby)) OR TI=(preadolescent)) OR AB=(preadolescent) OR TI=(toddler) OR AB=(toddler) OR TI=(youth) OR AB=(youth) OR TI=(juvenile) OR AB=(juvenile) OR TI=(newborn) OR AB=(newborn) OR TI=(newborns) OR AB=(newborns))))))) | 2223971 |
|  | **#2** | ((TI=(sars-cov-2) OR TI=(coronavirus) OR TI=(covid-19) OR TI=(severe acute respiratory syndrome coronavirus) OR TI=(covid) OR TI=(2019-n cov) OR ((AB=(sars-cov-2) OR AB=(coronavirus) OR AB=(covid-19) OR AB=(severe acute respiratory syndrome coronavirus) OR AB=(covid) OR AB=(2019-n cov))))) | 363988 |
|  | **#3** | (((TI=(MIS-C) OR TI=(Multisystem inflammatory syndrome)OR TI=(hyperinflammatory syndrome) OR TI=(inflammatory multisystem syndrome) OR TI=(multisystem inflammatory syndrome) OR TI=(Kawasaki‐like disease) OR TI=(Kawasaki Disease) OR TI=(hyperinflammatory syndrome) OR TI=(Multisystem inflammat*) OR (AB=(MIS-C) OR AB=(Multisystem inflammatory syndrome)OR AB=(hyperinflammatory syndrome) OR AB=(inflammatory multisystem syndrome) OR AB=(multisystem inflammatory syndrome) OR AB=(Kawasaki‐like disease) OR AB=(Kawasaki Disease) OR AB=(hyperinflammatory syndrome) OR AB=(Multisystem inflammat*))))) | 13092 |
|  | **#4** | (TI=(Circulatory Failure) OR TI=(Circulatory Collapse) OR TI=(shock)) OR (AB=(Circulatory Failure) OR AB=(Circulatory Collapse) OR AB=(shock)) | 316605 |
|  | **#5** | #1 AND #2AND #3 AND #4 | 303 |

**Table S3.** JBI tool for the quality assessment of studies

| **Study** | **Q1** | **Q2** | **Q3** | **Q4** | **Q5** | **Q6** | **Q7** | **Q8** | **Q9** | **Final JBI** |
| --- | --- | --- | --- | --- | --- | --- | --- | --- | --- | --- |
| Abdelaziz et al. | Yes | Yes | Moderate | Yes | Yes | Yes | Yes | Yes | Yes | Moderate |
| Abrams et al. | Yes | Yes | Yes | Yes | Yes | Yes | Yes | Yes | Yes | High |
| Acevedo et al. | Yes | Yes | Yes | Yes | Yes | Yes | Yes | Yes | Yes | High |
| Acharyya et al. | Yes | No | No | Yes | Yes | Yes | Yes | Yes | Yes | Moderate |
| Al Maskari et al. | Yes | No | No | Yes | Yes | Yes | Yes | Yes | Yes | Moderate |
| Alam et al. | Yes | Yes | Yes | Yes | Yes | Yes | Yes | Yes | Yes | High |
| Almoosa et al. | Yes | No | Moderate | Yes | Yes | Yes | Yes | Yes | Yes | Moderate |
| Suresh Kumar et al. | Yes | Yes | Yes | Yes | Yes | Yes | Yes | Yes | Yes | High |
| Avrusin et al. (2023 IRD) | Yes | Moderate | Moderate | Yes | Yes | Yes | Yes | Yes | Yes | Moderate |
| Avrusin et al. (Children) | Yes | Yes | Yes | Yes | Yes | Yes | Yes | Yes | Yes | High |
| Avrusin et al. (Biomedicines) | Yes | Yes | Yes | Yes | Yes | Yes | Yes | Yes | Yes | High |
| Awasthi et al. | Yes | Yes | Moderate | Yes | Yes | Yes | Yes | Yes | Yes | Moderate |
| Bagri et al. | Yes | Yes | Yes | Yes | Yes | Yes | Yes | Yes | Yes | High |
| Slöcker Barrio et al. | Yes | Yes | Yes | Yes | Yes | Yes | Yes | Yes | Yes | High |
| Bautista-Rodriguez et al. | Yes | Yes | Yes | Yes | Yes | Yes | Yes | Yes | Yes | High |
| Belay et al. | Yes | Yes | Yes | Yes | Yes | Yes | Yes | Yes | Yes | High |
| Belhadjer et al. | Yes | Moderate | Moderate | Yes | Yes | Yes | Yes | Yes | Yes | Moderate |
| Benvenuto et al. | Yes | Yes | Moderate | Yes | Yes | Yes | Yes | Yes | Yes | Moderate |
| Bichali et al. (2023) | Yes | Yes | Moderate | Yes | Yes | Yes | Yes | Yes | Yes | Moderate |
| Bichali et al. (2024) | Yes | Yes | Moderate | Yes | Yes | Yes | Yes | Yes | Yes | Moderate |
| Borgi et al. | Yes | No | No | Yes | Yes | Yes | Yes | Yes | Yes | Moderate |
| Campos et al. | Yes | Yes | Moderate | Yes | Yes | Yes | Yes | Yes | Yes | Moderate |
| Carbajal et al. | Yes | Yes | Yes | Yes | Yes | Yes | Yes | Yes | Yes | High |
| Caro-Domínguez et al. | Yes | Yes | Moderate | Yes | Yes | Yes | Yes | Yes | Yes | Moderate |
| Cattalini et al. | Yes | Yes | Yes | Yes | Yes | Yes | Yes | Yes | Yes | High |
| Chandran et al. | Yes | No | Moderate | Yes | Yes | Yes | Yes | Yes | Yes | Moderate |
| Chattopadhyay et al. | Yes | Moderate | Moderate | Yes | Yes | Yes | Yes | Yes | Yes | Moderate |
| Cheung et al. | Yes | No | No | Yes | Yes | Yes | Yes | Yes | Yes | Moderate |
| Chinniah et al. | Yes | Yes | Yes | Yes | Yes | Yes | Yes | Yes | Yes | High |
| Chiotos et al. | Yes | No | No | Yes | Yes | Yes | Yes | Yes | Yes | Moderate |
| Davies et al. | Yes | Yes | Yes | Yes | Yes | Yes | Yes | Yes | Yes | High |
| Dhooria et al. | Yes | Moderate | Moderate | Yes | Yes | Yes | Yes | Yes | Yes | Moderate |
| Elilarasi et al. | Yes | Moderate | Moderate | Yes | Yes | Yes | Yes | Yes | Yes | Moderate |
| de Farias et al. | Yes | Moderate | Moderate | Yes | Yes | Yes | Yes | Yes | Yes | Moderate |
| García Domínguez et al. | Yes | Moderate | Moderate | Yes | Yes | Yes | Yes | Yes | Yes | Moderate |
| García-Salido et al. | Yes | Yes | Yes | Yes | Yes | Yes | Yes | Yes | Yes | High |
| Grama et al. | Yes | Moderate | Moderate | Yes | Yes | Yes | Yes | Yes | Yes | Moderate |
| Gupta et al. | Yes | No | Moderate | Yes | Yes | Yes | Yes | Yes | Yes | Moderate |
| Jain et al. | Yes | Moderate | Moderate | Yes | Yes | Yes | Yes | Yes | Yes | Moderate |
| Jaxybayeva et al. | Yes | Yes | Moderate | Yes | Yes | Yes | Yes | Yes | Yes | Moderate |
| Khoury et al. | Yes | Yes | Yes | Yes | Yes | Yes | Yes | Yes | Yes | High |
| Kostik et al. | Yes | Yes | Yes | Yes | Yes | Yes | Yes | Yes | Yes | High |
| Lampidi et al. | Yes | Yes | Yes | Yes | Yes | Yes | Yes | Yes | Yes | High |
| Lee et al. | Yes | Moderate | Moderate | Yes | Yes | Yes | Yes | Yes | Yes | Moderate |
| Legarda et al. | Yes | Moderate | Moderate | Yes | Yes | Yes | Yes | Yes | Yes | Moderate |
| Lima-Setta et al. | Yes | Yes | Yes | Yes | Yes | Yes | Yes | Yes | Yes | High |
| Lopez et al. | Yes | No | No | Yes | Yes | Yes | Yes | Yes | Yes | Moderate |
| Maggio et al. | Yes | Moderate | Moderate | Yes | Yes | Yes | Yes | Yes | Yes | Moderate |
| Maheshwari et al. | Yes | Moderate | Moderate | Yes | Yes | Yes | Yes | Yes | Yes | Moderate |
| Mahmoud et al. | Yes | Yes | Yes | Yes | Yes | Yes | Yes | Yes | Yes | High |
| Mannarino et al. | Yes | No | No | Yes | Yes | Yes | Yes | Yes | Yes | Moderate |
| Mehra et al. | Yes | Yes | Yes | Yes | Yes | Yes | Yes | Yes | Yes | High |
| Menchaca-Aguayo et al. | Yes | Yes | Yes | Yes | Yes | Yes | Yes | Yes | Yes | High |
| Miller et al. | Yes | Yes | Yes | Yes | Yes | Yes | Yes | Yes | Yes | High |
| Nadua et al. | Yes | No | No | Yes | Yes | Yes | Yes | Yes | Yes | Moderate |
| Nayak et al. | Yes | Yes | Yes | Yes | Yes | Yes | Yes | Yes | Yes | High |
| Niño-Taravilla et al. | Yes | No | No | Yes | Yes | Yes | Yes | Yes | Yes | Moderate |
| Pawar et al. | Yes | No | No | Yes | Yes | Yes | Yes | Yes | Yes | Moderate |
| Rekhtman et al. | Yes | Yes | No | Yes | Yes | Yes | Yes | Yes | Yes | Moderate |
| Renson et al. | Yes | Yes | Yes | Yes | Yes | Yes | Yes | Yes | Yes | High |
| Sen et al. | Yes | No | No | Yes | Yes | Yes | Yes | Yes | Yes | Moderate |
| Shahbaznejad et al. | Yes | No | No | Yes | Yes | Yes | Yes | Yes | Yes | Moderate |
| Shobhavat et al. | Yes | No | No | Yes | Yes | Yes | Yes | Yes | Yes | Moderate |
| Sperotto et al. | Yes | Yes | Yes | Yes | Yes | Yes | Yes | Yes | Yes | High |
| Toubiana et al. | Yes | Yes | Moderate | Yes | Yes | Yes | Yes | Yes | Yes | Moderate |
| Valverde et al. | Yes | Yes | Yes | Yes | Yes | Yes | Yes | Yes | Yes | High |
| Varadarajan et al. | Yes | Yes | Yes | Yes | Yes | Yes | Yes | Yes | Yes | High |
| Varga et al. | Yes | Yes | Moderate | Yes | Yes | Yes | Yes | Yes | Yes | Moderate |
| Whittaker et al. | Yes | Yes | Yes | Yes | Yes | Yes | Yes | Yes | Yes | High |
| Yilmaz Ciftdogan et al. | Yes | Yes | Yes | Yes | Yes | Yes | Yes | Yes | Yes | High |

**Table S4.** Quality assessment of studies with the ROBINS-E for risk assessment

| **Study** | **Confounding** | **Exposure Measurement** | **Selection of Participants** | **Post-Exposure Interventions** | **Missing Data** | **Outcome Measurement** | **Selection of Reported Result** | **Overall ROBINS-E Judgment** |
| --- | --- | --- | --- | --- | --- | --- | --- | --- |
| García-Salido et al. (2020) | Moderate | Moderate | Low–Moderate | Moderate | Moderate | Low | Moderate | Moderate risk |
| García-Domínguez et al. (2023) | Serious | Moderate | Moderate | Moderate | Moderate | Low | Moderate | Serious risk |
| Farias et al. (2023) | Moderate | Low | Moderate | Moderate | Low–Moderate | Low | Moderate | Moderate risk |
| Gupta et al. (2021) | Serious | Low | Serious | Moderate | Moderate | Low | Moderate | Serious risk |
| Angurana et al. (2021) | Moderate | Low | Serious | Moderate | Moderate | Low | Moderate | Serious risk |
| Legarda et al. (2023) | Moderate | Low | Serious | Moderate | Moderate | Low | Moderate | Serious risk |
| Khoury et al. (2023) | Moderate | Low | Moderate | Moderate | Moderate | Low | Moderate | Moderate risk |

**Table S5: Meta-regression**

| **Covariate** | **Univariable β (95% CI)** | **Univariable p-value** | **Multivariable β (95% CI)** | **Multivariable p-value** |
| --- | --- | --- | --- | --- |
| Year of publication | -0.0797 (-0.1371 to -0.0223) | 0.0072 | -0.0671 (-0.1378 to 0.0036) | 0.0625 |
| Mean/median age | -0.0155 (-0.0505 to 0.0194) | 0.3769 | -0.0064 (-0.0459 to 0.0330) | 0.7441 |
| Male proportion (%) | -0.0038 (-0.0094 to 0.0017) | 0.1744 | -0.0035 (-0.0095 to 0.0025) | 0.2477 |
| Sample size (N) | Not assessed | — | -0.0000 (-0.0001 to 0.0001) | 0.8652 |
| Diagnostic criteria | Overall moderator p = 0.2223 | 0.2223 | Overall moderator p = 0.2657 | 0.2657 |

## **Table S6. GRADE assessment for prevalence and comparative risk outcomes**

| **Outcome** | **No. of Studies** | **Study Design** | **Risk of Bias** | **Inconsistency** | **Indirectness** | **Imprecision** | **Publication Bias** | **Other Considerations** | **Overall Certainty of Evidence** |
| --- | --- | --- | --- | --- | --- | --- | --- | --- | --- |
| Prevalence of shock among children with MIS-C | 70 studies | Observational studies | Serious | Very serious | Not serious | Not serious | Undetected | Large pooled sample size (>13,000 participants) | Very Low ⨁◯◯◯ |
| Risk of shock in MIS-C versus Kawasaki disease | 5 studies | Observational comparative studies | Serious | Serious | Not serious | Serious | Undetected | Large magnitude of effect (RR > 8) | Very Low ⨁◯◯◯ |
| Risk of shock in MIS-C versus acute/severe COVID-19 | 7 studies | Observational comparative studies | Serious | Moderate | Not serious | Not serious | Undetected | Large magnitude of effect (RR > 4) | Low ⨁⨁◯◯ |
| Predictors of shock in MIS-C (narrative synthesis) | Multiple observational studies | Observational studies | Serious | Serious | Not serious | Serious | Suspected | Inability to perform quantitative pooling | Very Low ⨁◯◯◯ |

**Figure S1:** Sensitivity analysis after excluding using studies less than 50 sample size

**Figure S2:** Sensitivity analysis after excluding using studies less than 100 sample size

**Figure S3:** Subgroup analysis by country

**Figure S4:** Subgroup analysis by diagnostic criteria

**Figure S5:** Subgroup analysis by year

**Figure S6:** Funnel plot after excluding studies less than 50 sample size

**Figure S7:** Funnel plot after excluding studies less than 100 sample size
